# Scoliosis Surgery in a Patient With Advanced Friedreich's Ataxia—It Is Not Too Late

**DOI:** 10.1002/acn3.70219

**Published:** 2025-10-03

**Authors:** Kathrin Reetz, Stella A. Lischewski, Jörg B. Schulz, Maximilian Praster, Miguel Pishnamaz, the FACROSS study group, Imis Dogan, Sandro Romanzetti, Ravi Dadsena, Kerstin Konrad, Thomas Clavel, Vera Jankowski, Joachim Jankowski, Oliver Pabst, Nikolaus Marx, Julia Moellmann, Malte Jacobsen, Katharina Marx‐Schütt, Juergen Dukart, Simon Eickhoff, Ralf‐Dieter Hilgers

**Affiliations:** ^1^ Department of Neurology RWTH Aachen University Aachen Germany; ^2^ JARA‐BRAIN Institute Molecular Neuroscience and Neuroimaging Research Centre Juelich GmbH and RWTH Aachen University Aachen Germany; ^3^ Department for Orthopaedics, Trauma and Reconstructive Surgery RWTH Aachen University Hospital Aachen Germany; ^4^ Section Child Neuropsychology, Department of Child and Adolescent Psychiatry, Psychosomatics and Psychotherapy RWTH Aachen, University Hospital Aachen Germany; ^5^ Functional Microbiome Research Group, Institute of Medical Microbiology RWTH University Hospital Aachen Germany; ^6^ Institute for Molecular Cardiovascular Research RWTH Aachen University Hospital Aachen Germany; ^7^ Institute of Molecular Medicine RWTH Aachen University Aachen Germany; ^8^ Department of Internal Medicine I, Cardiology RWTH Aachen University Aachen Germany; ^9^ Institute of Neuroscience and Medicine, Brain and Behaviour (INM‐7), RResearch Centre Jülich Jülich Germany; ^10^ Institute of Systems Neuroscience Medical Faculty, Heinrich Heine University Düsseldorf Düsseldorf Germany; ^11^ Department of Medical Statistics RWTH Aachen University Aachen Germany

**Keywords:** Friedreich ataxia, quality of life, scoliosis, surgery

## Abstract

Friedreich's ataxia is a multisystem disorder with scoliosis being the most common non‐neurological manifestation. While scoliosis surgery is typically performed in adolescent, ambulatory patients, few data exist on surgical outcomes in patients with advanced disease. We present a 38‐year‐old woman with late‐stage Friedreich's ataxia and pronounced thoracolumbar scoliosis (Cobb angle 48°) causing severe pain and limited sitting tolerance. After posterior corrective spondylodesis (T4‐ilium), she reported marked improvements in pain, sitting tolerance, function, and quality of life in the SF‐36 questionnaire. This case highlights the potential for substantial clinical and functional benefits from scoliosis surgery in patients with advanced Friedreich's ataxia.

## 1 Introduction

Friedreich's ataxia is a multisystem neurodegenerative disorder. Scoliosis, the most prevalent non‐neurological symptom in Friedreich's ataxia, affects 63%–90% [1, 2, 3, 4, 5] of patients and frequently emerges before the onset of ataxia [2]. Scoliosis in Friedreich's ataxia primarily results from neuromuscular weakness, leading to impaired trunk stabilization. In advanced stages, this can cause patients to collapse in their wheelchairs. Beyond back pain, scoliosis can affect cardiopulmonary function, ambulation, and activities of daily living, ultimately compromising quality of life.

Treatment options comprise conservative management with physiotherapy or braces and surgery. Surgical procedures typically involve spinal fusion to achieve a straightened spine and balanced pelvis [6]. Indications include severe scoliosis with a Cobb angle > 45°, depending on the type of curve and muscular stability, and a reduction in vital capacity by 50% [7, 8]. Most scoliosis surgeries in Friedreich's ataxia are performed early in the disease course, often in ambulatory patients [1] and, to our knowledge, few data are available on outcomes in patients with advanced disease. This report presents the case of a patient with advanced Friedreich's ataxia who experienced significant improvements following scoliosis surgery, with a 4‐year follow‐up.

## 2 Case Report

A 38‐year‐old woman with genetically confirmed Friedreich's ataxia (onset at age 13 years) presented to our ataxia clinic with severe back pain secondary to scoliosis, with recent progression on clinical examination. She was wheelchair‐bound, unable to sit for more than 2 h per day due to pain and reported low mood.

**FIGURE 1:**
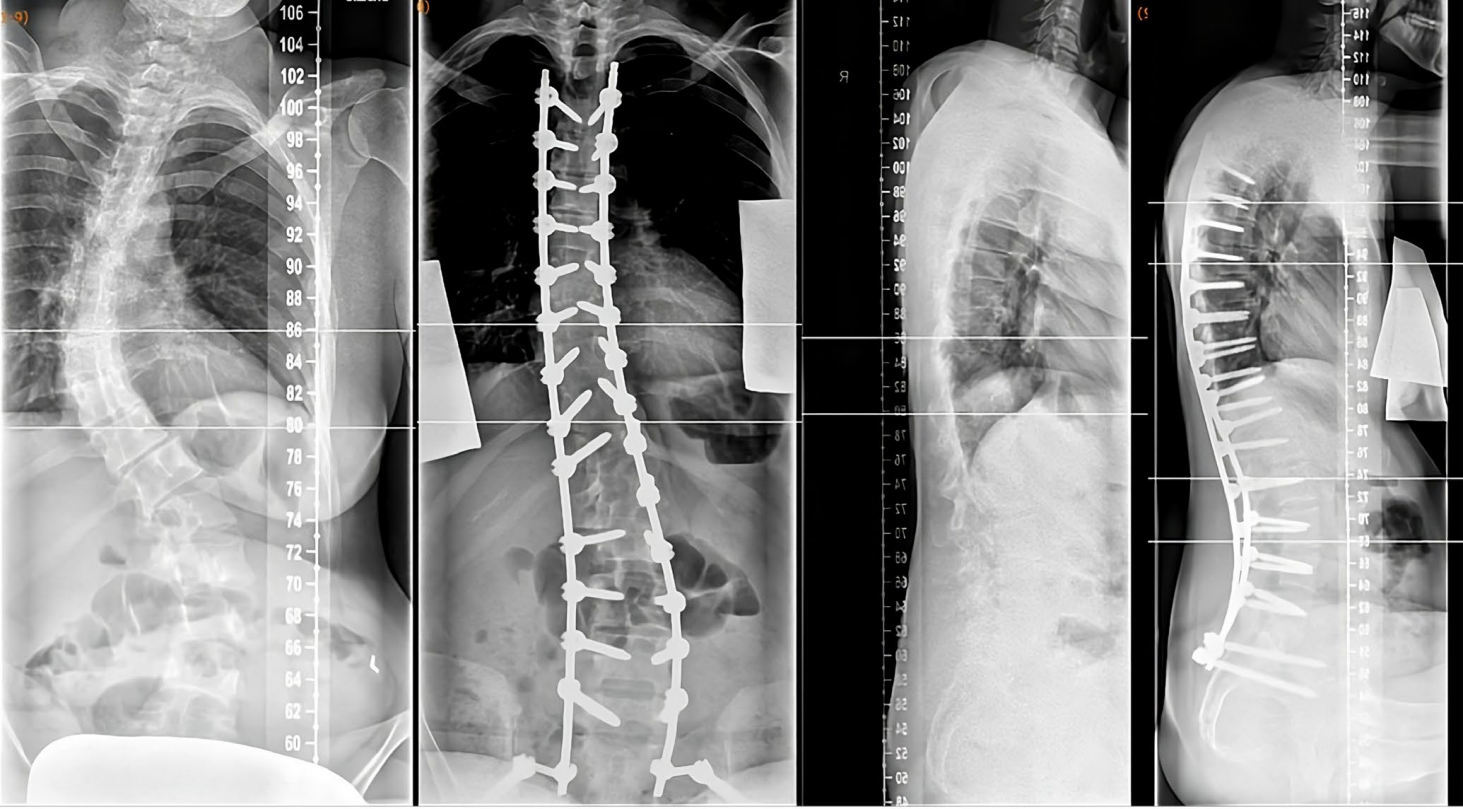
S‐shaped thoraco‐lumbar scoliosis. The preoperative x‐ray spinal images showed the right convex part of the thoracic spine with a Cobb angle of c. 52° and left convex counter swing of the lumbar spine with a Cobb angle of c. 48°. One year post surgery the spinal x‐ray images showed a thoracic Cobb angle of c. 24° and lumbar 15°.

Neurological examination revealed severe ataxia, lower limb weakness, and spasticity. Spine examination demonstrated severe scoliosis, with contact between the right costal arch and the iliac crest, impairing trunk stability. Imaging (in sitting position) showed a pronounced S‐shaped thoracolumbar scoliosis: a right‐convex thoracic curve (Cobb angle 52°, T6–T11) with mild vertebral rotation (Grade 1 Nash and Moe), and a left‐convex lumbar counter curve (Cobb angle 48°, T12–L4) with severe rotation (Grade 3, Nash and Moe). There was a significant shift of the central sacral vertical line to the left with resulting trunk shift to the right (Figure 1). Spinal MRI excluded additional abnormalities. Cardiac MRI revealed mild left ventricular hypertrophy with normal ejection fraction.

**FIGURE 2:**
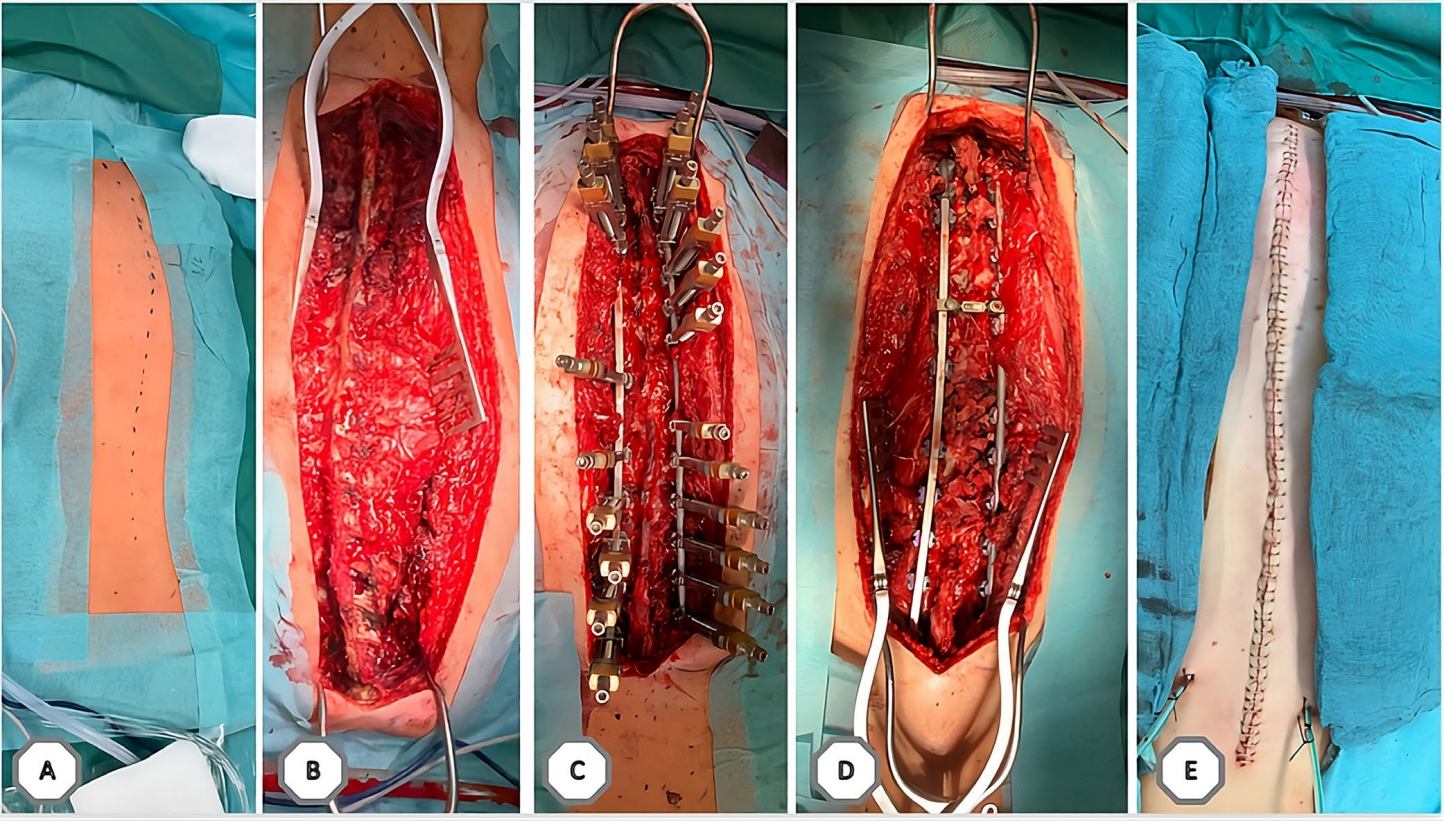
Typical intraoperative situation and procedure. (A) The dotted line delineates the contour of the spine on the skin. The patient is positioned in the prone posture. (B) Open situs is conducted to visualize the spine. (C) Implementation of the rig and correction system to straighten the spine. (D) Final outcome of spinal correction within the body. (E) Surgical wound is sutured for closure.

Initial conservative management with analgesia failed to relieve her pain. Outweighing the risks and benefits and upon multidisciplinary consensus, a posterior corrective spondylodesis from T4 to the iliac bone was performed (analog to the typical procedure outlined in Figure 2). Postoperatively, the patient experienced a short episode of respiratory distress requiring re‐intubation and intensive care. However, she stabilized quickly and was soon discharged to the ward. Subsequent recovery was uneventful, with successful weaning of analgesics and normal spinal alignment. She was discharged 12 days post‐surgery and continued with regular outpatient physiotherapy.

At follow‐up, the patient reported significant pain relief, enabling her to sit in her wheelchair for 8–10 h daily, and improved swallowing. Her SF‐36 Physical Component Summary Score (0–100 scale, normed to a mean of 50) increased from 15.7 pre‐op to 32.3 post‐op, and the SF‐36 Mental Component Summary Score (0–100 scale, normed to a mean of 50) from 31.9 to 36.7 on 4‐year follow‐up, indicating improved quality of life. The Activities of Daily Living score quantifying functional impairment [9] remained unchanged postoperatively (25/36 points). She further reports being able to sit for extended periods. Radiographic follow‐up demonstrated marked scoliosis correction with harmonization of the trunk shift and excellent sagittal alignment (Figure 1). However, a slight elevation of the left shoulder without clinical relevance was observed.

## 3 Discussion

Our patient had a 25‐year disease duration and was wheelchair‐bound. Previous literature suggests that scoliosis surgery in Friedreich's ataxia is rarely performed after the age of 20 years [1, 2, 3, 4] and mostly in ambulatory individuals [3, 5]. This trend may be attributed to slower scoliosis progression past adolescence, heightened concerns about cardiopulmonary complications, and the benefits of preserving upright stability in ambulatory patients [6]. However, nonambulatory patients can be considered for fusion to the pelvis without compromising mobility, reducing revision surgery risk.

Preoperative concerns in our patient included cardiopulmonary complications exacerbated by hypertrophic cardiomyopathy, dysphagia, and anticipated blood loss due to prolonged operation time. Despite requiring re‐intubation, the patient stabilized quickly. Perioperative mortality in patients with Friedreich's ataxia has, to our knowledge, been documented in one case involving a 9‐year‐old girl with left‐ventricular dysfunction in 1978 [4] and in 0.2% of cases of patients with other neuromuscular diseases [10]. Furthermore, a case series in patients with Friedreich's ataxia reported that 1 of 16 patients required brief intensive care monitoring due to respiratory distress [3]. This underscores the importance of comprehensive preoperative evaluation, particularly in patients like ours with advanced Friedreich's ataxia, to identify and mitigate potential perioperative risks. A detailed cardiopulmonary risk‐assessment including echocardiography and ECG to assess the risk of anesthesia is imperative, especially in patients with preexisting hypertrophic cardiomyopathy.

Fortunately, our patient experienced no significant postoperative complications. Although published data on postoperative complications in patients with Friedreich's ataxia remain limited and heterogeneous, they suggest a relatively frequent occurrence. In three small case series, wound infection rates ranged from 0% [4] to 11.1% [2], while implant failure occurred in 6.3% [3] to 66.7% of patients, highlighting a particularly high mechanical complication rate in one cohort [2]. Proximal junctional kyphosis was reported in 6.3% [3] to 23.5% [4] of cases, and pseudoarthrosis in up to 5.9% [4]. These findings are broadly consistent with complication patterns seen in the wider neuromuscular scoliosis population, where overall complication rates approach 38% [10]. The most frequently reported issues include wound‐related complications (13%), respiratory events (12%), and revision surgeries (10%), often for wound or implant‐related problems. Additional complications include implant‐related failures (7%), gastrointestinal dysfunction (5%), pseudoarthrosis (5%), and neurological injury (3%) [10]. While our case had a favorable outcome, this should not be assumed to be representative. Given the potential risks, patients with Friedreich's ataxia should be carefully counseled on the likelihood of surgical complications, and it should be acknowledged that, in some cases, the risks of operative intervention may outweigh the anticipated benefits.

In conclusion, this case exemplifies the potential benefits of scoliosis surgery in patients with Friedreich's ataxia in later disease stages. The patient reported significant improvement in pain, quality of life, and functional ability, highlighting the importance of patient‐reported outcomes, which, to our knowledge, are underrepresented in existing studies.

## Author Contributions

All authors conceived the case report. K.R., S.A.L. and M.Pi. were involved in the clinical care of the patient. S.A.L. analyzed the SF‐36 and ADL data. K.R. and S.A.L. wrote a draft of the manuscript. All authors reviewed and edited the manuscript.

## Ethics Statement

The patient has given written and informed consent for the publication of this case report. Review of an ethics committee was not required for this work.

## Conflicts of Interest

K.R. has received grants from the German Research Foundation, Friedreich's Ataxia Research Alliance (FARA), Interdisciplinary Center for Clinical Research within the faculty of Medicine at the RWTH Aachen University, Germany (OC2), German Federal Ministry of Education and Research (BMBF KP22‐106E), and honoraria for presentations or advisory boards from Biogen, Eisai, Lilly, and Roche. S.A.L. has received speaking and advisory honoraria from Biogen. J.B.S. received grants from the German Research Foundation, Interdisciplinary Center for Clinical Research within the faculty of Medicine at the RWTH Aachen University, Germany, Biogen, and Eisai, and for presentations or advisory boards from Biogen, Reata, Eisai, Lilly, Roche, and NovoNordisk. M.Pr. and M.Pi. declare no conflicts of interest.

## Data Availability

The data that support the findings of this study are available from the corresponding author upon reasonable request.
